# Sociodemographic correlates of perceived COVID-19 impact on movement behaviors in Croatian school-aged children

**DOI:** 10.1186/s12982-026-02827-9

**Published:** 2026-10-07

**Authors:** Tamara Balja, Helena Križan, Slaven Krtalić, Sanja Musić Milanović

**Affiliations:** 1 Division for Health Promotion, Croatian Institute of Public Health, Zagreb, 10000 Croatia; 2 School of Public Health “Andrija Štampar”, University of Zagreb School of Medicine, Zagreb, 10000 Croatia

**Keywords:** Children, Physical activity, Sedentary behavior, Sleep, COVID-19

## Abstract

**Background:**

Levels of physical activity (PA), sedentary behavior (SB), and sleep, as important determinants of children’s health and weight status, may have been significantly affected by the COVID-19 pandemic. This cross-sectional study aimed to determine factors associated with parental perception of COVID-19 negative impact in PA, SB, and sleep during the COVID-19 period, and whether the parental perception of COVID-19 negative impact was associated with actual post-lockdown behavioral levels among 2nd and 3rd grade elementary school students in Croatia.

**Method:**

Data was gathered on a nationally representative sample of Croatian children participating in the CroCOSI 2021/2022 study. The final sample consisted of 5438 children (male = 51.4%, female = 48.6%) aged 7 to 10 years (*M* = 9.03, *SD* = 0.61). Risk and protective factors included children’s weight status, demographics, and socioeconomic status (SES) variables. Parents completed the paper surveys on children’s actual post-lockdown behavioral levels in PA, SB and sleep, and the perceived COVID-19 negative impact on these behaviors.

**Results:**

Multinomial logistic regression models showed that the parental perception of negative COVID-19 impact on active play, screen time, and learning time was associated with various SES indicators, child-related variables, and degree of urbanization. Parents with high education levels were more likely to perceive a negative impact across all behaviors. Children of parents with high education compared to low had a higher likelihood of parental perception of COVID-19 negative impact on screen time (weekdays: *OR* = 0.48; CI = 0.40, 0.56; weekend: *OR* = 0.59; CI = 0.50, 0.70), learning time (*OR* = 0.49; CI = 0.41, 0.58), and active play (weekdays: *OR* = 0.57; CI = 0.48, 0.68; weekend: *OR* = 0.71; CI = 0.59, 0.86), but also to a higher likelihood of parents perceiving positive changes in active play. On the other hand, lower perceived family wealth increased the odds of parental perception of COVID-19 negative impact on active play (weekdays: *OR* = 1.40; CI = 1.08, 1.81; weekends: *OR* = 1.42; CI = 1.10, 1.82) and screen time (weekdays: *OR* = 1.36; CI = 1.07, 1.74; weekends: *OR* = 1.51; CI = 1.19, 1.91), while on weekends it was also linked to perceived positive changes in screen time (*OR* = 2.07; CI = 1.11, 3.83). Regarding the second study aim, complex samples of general linear models showed that the parental perception of negative COVID-19 impact was significantly associated with actual post-lockdown active play and screen time levels. A parental perception of COVID-19 negative impact on active play corresponded to about 12 min less active play per weekday and weekend day post-lockdown, while a parental perception of increased screen time was associated with about 12 min more screen time per weekday and 28 min more screen time per weekend day post-lockdown.

**Conclusion:**

The study results suggest that the parental perception of COVID-19 impact on children’s movement behaviors varied according to sociodemographic factors of the children and their families, and was also associated with certain post-lockdown movement behavior in children. These findings highlight the need for targeted public health strategies that provide safe and accessible opportunities for play and physical activity while reducing excessive screen time. Continuous multisectoral efforts are important for promoting healthy lifestyles and preventing childhood obesity.

**Supplementary Information:**

The online version contains supplementary material available at https://doi.org/10.1186/s12982-026-02827-9.

## Introduction

The rise in childhood obesity is a significant public health challenge of the 21st century. Over the past four decades, the prevalence of overweight and obesity in children and adolescents has significantly increased worldwide [1–3], with the estimation that in 2022 more than 390 million children and adolescents worldwide suffered from overweight, including obesity [3]. Obesity arises from a complex interplay of many factors, including genetic, socioeconomic, environmental, and behavioral characteristics, with physical activity (PA), sedentary behavior (SB), and sleep being important modifiable components in maintaining health and healthy weight status [4–6]. Engaging children in regular PA is of vital significance, given its well-documented health benefits and important role in preventing obesity [7–9]. According to the World Health Organization (WHO), children aged 5–17 should engage in, on average, at least 60 min of moderate-to-vigorous PA per day [10]. Children can meet the recommended PA guidelines through active travel, participation in sports and dance activities, and active play [11]. Alongside the protective effects of regular PA, excessive SB is associated with an increased risk of obesity in children [10, 12–14]. SB is described as a behavior that occurs while sitting, reclining, or lying down during awake time and is characterized by very low energy expenditure, of ≤ 1.5 metabolic equivalents [10, 15]. Primarily through time spent using screen-based devices, prolonged SB in school-aged children is associated with unfavorable body composition and lower fitness, as well as some other outcomes, for example, lower self-esteem and unfavorable social behavior [12, 13, 16]. Short sleep duration has also been associated with an increased risk of overweight/obesity in children [17–19]. Influencing obesity through several pathways [20], it can increase caloric intake, affecting the hormones ghrelin (which stimulates appetite) and leptin (which signals satiety) [21, 22], and may also lead to fatigue and tiredness, leading to decreased PA and increased sedentary activity [23].

### COVID-19

During the COVID-19 pandemic, authorities implemented various measures, including self-isolation, school closures, lockdowns, and social distancing guidelines, to reduce the spread of the virus. In Croatia, as part of the COVID-19 containment measures, the government implemented school closures throughout the country, starting on 16 March 2020. Shortly after (in the following month) schools reopened for younger children to be able to attend in-person classes, according to guidelines and recommendations by Croatian Institute of Public Health (CIPH), with temporary school closings in the following period, when the epidemiological situation would require this [24]. Containment measures disrupted family lifestyles worldwide and altered children’s daily routines, and, as opportunities for regular PA were limited, maintaining PA became very challenging [25, 26]. Findings from the COSI-COVID study conducted across 17 countries in the WHO European Region indicate negative impacts of the pandemic on children’s movement behavior, including an almost 30% decrease in weekday PA levels and an increase in screen time duration among one-third of children [25]. Given these observed effects, studying changes in children’s movement behavior and their possible lasting effects is of significant importance, especially in the context of the ongoing trends in childhood obesity [25, 27]. Early studies have shown the immediate changes in children’s movement behavior, in terms of decreased PA, increased SB, and disrupted sleep schedule due to COVID-19-related restrictions [28–30]. Results from a longitudinal study indicate that the median time spent in PA in children and adolescents decreased from 540 min per week (before the pandemic) to 105 min per week (during the pandemic), while screen time significantly increased (approximately 30 h per week on average) [29]. The Dutch study which included both self-reported and objectively measured PA via accelerometry, as well as screen time duration among children, found that the number of children meeting the recommended PA guidelines of ≥ 60 min per day decreased from 64% to 20% during the pandemic. Screen time duration during the pandemic was higher compared to the time pre-school closures, even after schools and sports clubs reopened, suggesting a lasting effect of COVID-19 on movement behavior [26]. A German study found an increase in screen time among children aged 4–17, and a decrease in sports activities. Interestingly, an increase in habitual activity was found, leading to the overall rise in PA in German children compared to the pre-lockdown period [31]. During the COVID-19 pandemic, changes in daily routines also significantly disrupted children’s sleep patterns. One systematic review found that the pooled prevalence of sleep disturbances during the COVID-19 pandemic in children, including children with neurobehavioral disorders, was 54%, and nearly three times as many children experienced poorer sleep quality during the pandemic than those who reported improvements [32]. The other two meta-analyses reported the prevalence of sleep disturbances in children and adolescents during the COVID-19 pandemic of 34% [33] and 44% [34]. On the other hand, COSI-COVID study findings indicate that, according to the parental reports, the sleep patterns remained the same for most children during the pandemic [25].

Some studies indicate that the impact of COVID-19 and associated measures may not have affected all children equally, depending on some child, family, and sociodemographic characteristics. An Australian study found that the children from higher socioeconomic backgrounds, as indicated by a neighborhood-level measure of income, experienced a greater increase in screen time compared to those from lower socioeconomic backgrounds [35], while a Swiss study found that children from disadvantaged households were more likely to experience changes in health behaviors, whether positive or negative [36]. A study conducted in Italy found that reduced time spent in active play, along with increased screen time and a more sedentary lifestyle, was observed among children who had at least one parent with a medium or high level of education and who came from families with lower perceived financial status. The decline in physical activity was particularly noticeable in households without access to outdoor spaces [37], consistent with findings from some other countries [38, 39]. An Indonesian study also showed that decreased family income during the COVID-19 pandemic was associated with greater risk for reduced physical activity among children [40]. Results from the study conducted in Portugal [41] which examined the role of household variables in children’s PA during the lockdown, younger age was the strongest predictor of PA, followed by having a large outdoor space, more children in the household, and at least one adult not working from home. In a study conducted in Germany, children aged 10–12 years and children from low-income families showed the greatest reduction in physical activity compared with younger and older age groups and children from higher-income families [42].

According to the Childhood Obesity Surveillance Initiative conducted in Croatia (CroCOSI) in the 2021/2022 school year, 36.5% of children during the pandemic spent less time in active/vigorous play outside school hours on weekdays, and 29.9% did so during weekends. Parental reports also indicated a considerable rise in screen use, with recreational screen time increasing by 39.5% on weekdays and 37.5% on weekends [43]. Since other research findings indicated the importance of sociodemographic factors in moderating the COVID-19 impact on children’s movement behavior, our study aims to determine the risk and protective factors associated with parental perception of COVID-19 impact in PA levels, SB, and sleep during the COVID-19 pandemic (COVID-19 impact) among 2nd and 3rd grade elementary school students in Croatia, included in the CroCOSI study. A greater perceived negative impact of the COVID-19 pandemic on children’s movement behaviours was expected among older children, those living in urban areas due to limited access to outdoor spaces during lockdown, and children from families with lower SES. Our study also aimed to determine whether parental perceptions of the negative impact of the COVID-19 pandemic on children’s movement behaviours were associated with post-lockdown PA, SB, and sleep levels. We hypothesized that parental perceptions of negative changes in children’s movement behaviours during the COVID-19 period would be associated with actual post-lockdown levels of PA, SB, and sleep after the restrictive measures had ended.

## Materials and methods

The Childhood Obesity Surveillance Initiative (COSI) was established by the WHO Regional Office for Europe (WHO/Europe) in 2007 to collect comparable data on children’s body weight, height, and hip and waist circumference, among children aged 6.0–9.9 years [44]. COSI Protocol [45] was defined by the WHO/Europe team so that every participating country could follow the exact instructions. This paper was based on the Croatian COSI study (CroCOSI) from 2021 to 2022 [43]. The data collection occurred between February 28 and April 13 2022, and followed the standardized WHO COSI Data collection procedures [45].

### Ethics

CroCOSI received ethical approval from the Croatian Institute for Public Health research Ethics Committee (Class: 030 − 02/18 − 07/2) and was conducted in accordance with the modern ethical principles, with respect for the fundamental bioethical principles of autonomy, justice, beneficence, and nonmaleficence. Informed consent for all study procedures was obtained from the children’s parents or legal guardians. The children were measured during school class time, and their parents voluntarily completed the paper surveys (COSI Family form) at home or during parents’ meetings at school, under the teacher’s supervision. Teachers were given instructions on administering the paper survey. The children also gave their assent before participating in the anthropometric measurements and were free to withdraw at any time without consequences.

### Sample

The sampling of Croatian children was conducted at a national level and in accordance with the COSI Protocol which specifies that the effective sample size—the number of measured children—should be at least 2800 children per target age group [45]. The initial sample was based on a single-stage cluster sampling method, where schools were stratified by region (Pannonian, Adriatic, Northern, and City of Zagreb). A 2nd-grade class was randomly selected, and a 3rd-grade class from the same school was matched to it, so that all children in those two classes were eligible to participate in the study. Post-stratification weights estimated by the WHO Regional Office for Europe were used in all analyses to infer the results from the sample to the population. The post-stratification weights (by stratum, i.e., the region where the school is located) are designed to adjust for the sampling design, oversampling, and non-response. Data on the total number of classes and students by stratum and grade that were used to calculate the weights were obtained from the Ministry of Science, Education, and Youth.

#### Missing data

In the CroCOSI 2021/2022 study [43], the family’s record form also included questions about the perceived impact of the COVID-19 pandemic on the child’s daily routine, well-being, dietary habits, and PA-related behaviors. Children whose parents did not fill in the family form at all, or did not fill in the additional COVID-19 form, were not included in the analysis. Only participants who completed at least one of the questions about the parental perception of COVID-19 impact on sleep, PA, and SB were included in the analysis. Of the 5603 participants in the study, 162 (2.9%) did not fill in at least one of the questions referenced above. The overall proportion of missing data was low (less than 5% for most of the examined variables), while seven variables had missing values between 5% and 8.4%. Missing values were handled pairwise, so cases were excluded only from analyses for which data were missing. No sensitivity analyses were conducted to assess potential bias introduced by missing data, as the percentage of missing data was relatively low. Ages 11–13 years were removed (*N* = 4) as they were considered outliers. The final weighted data set consisted of 5438 children (male = 51.4%, female = 48.6%) aged 7 to 10 years and 11 months (*M* = 9.03, *SD* = 0.61).

### Variables

#### Risk and protective factors

The parents were asked to identify their child’s gender as male (1) or female (2). This information was also checked against data from the COSI Child’s form, obtained from official school records. The parents also wrote in their child’s date of birth. This data was also checked against the data in the Child’s form and used to calculate the child’s age, in combination with the date the child’s measurements were taken. The age was calculated using the following formula: Age in years = (date of measurement-date of birth)/365.25. Data on the child’s weight status were derived from anthropometric measurements taken by trained examiners (weight in kg and height in cm). All measurements were taken using standardized equipment and in line with standard COSI Data collection procedures [45]. Children’s weight status was classified according to the 2007 WHO-recommended growth reference for school-aged children and adolescents [46, 47]. Thinness is defined as a BMI-for-age value < -2 Z-score, normal weight as BMI-for-age between − 2 and + 1 Z-score, and overweight and obesity are defined as a BMI-for-age value > + 1 Z-score and > + 2 Z-score, respectively [48].

Items from the COSI Family form were used to assess parent-reported socioeconomic status (SES). Items included (a) education level of the person filling out the questionnaire, (b) education level of the partner, (c) employment status of the person filling out the questionnaire, (d) employment status of the partner, and (e) perceived financial situation. In combination with information on who the person filling out the questionnaire was (i.e., mother, father, or someone else) and who is living with the child in the household, information on the education level and employment status of mothers and fathers was derived. Following the COSI methodology [43, 45], three categories of parental education were created: (i) low, where both parents have lower education (i.e. “primary school or less,” “secondary or high school,” or “vocational school”); (ii) medium, where one parent has lower education and the other has higher education (i.e. “undergraduate or bachelor’s degree” and “master’s degree or higher”); and (iii) high, where both parents have higher education. Two categories of parents’ employment status were created: (i) low (one or more parent(s) unemployed or economically inactive, i.e., not working at all and neither available nor looking for work); and (ii) high (both parents employed). Three categories of family-perceived wealth were created: (i) low family-perceived wealth (those who barely make ends meet each month with their earnings or have trouble making ends meet each month with their earnings); (ii) medium family-perceived wealth (those who pass the month without serious problems with their earnings) and; (iii) high family-perceived wealth (those who easily pass the month with their earnings).

Information about the children’s place of residence was also obtained from official school records. In line with the EUROSTAT classification of local administrative units, the researchers have categorized the child’s place of residence as urban (city, town/suburb) or rural area [49]. The schools were also grouped into four regions, according to the NUTS-2 classification: Pannonian Croatia, Adriatic Croatia, City of Zagreb, and Northern Croatia.

#### Parental perception of COVID-19 impact on sleep, PA and SB

Questions on parental perception of COVID-19 impact on children’s movement behavior are part of the COSI COVID-19 form used in 17 countries as part of the COSI in 2021–2023 [25]. Other questions in the COSI COVID-19 form address the impact of COVID-19 on children’s daily routines, well-being, and dietary habits; however, since this paper is focused on movement behaviors, only the questions investigating parental perceptions of COVID-19’s impact on children’s sleep, PA, and SB will be analyzed. In the COSI COVID-19 form, parents were instructed to select the months during which their child was home from school because of COVID-19 pandemic, between 1 March 2020 and the day data collection was conducted. Among parents who provided information about the period when their child was at home because of COVID-19 (*N* = 4641), the months during which the largest proportion of parents reported their child being home were March 2020 (44.84%), April 2020 (48.59%), May 2020 (37.51%) and December 2020 (25.62%), as well as November 2021 (31.35%), December 2021 (22.13%), January 2022 (53.29%) and February 2022 (19.67%). Parents were instructed that, for the purposes of the study, the period before 1 March 2020 was defined as “pre-COVID”, and the “COVID 19 pandemic period” the time during which their child was home due to COVID-19. This definition reflected the period that parents experienced as the lockdown, during which their child stayed at home, as the timing of COVID-19 restrictions varied across different regions. The survey question on parental perception of COVID-19 impact on children’s movement behavior was phrased as follows: “During the COVID-19 pandemic period indicates whether YOUR CHILD´s life changed in comparison to the pre-COVID period, and please select for each situation if the issue described decreased, stayed the same or increased when compared to the pre-COVID19 period”. Parents were asked to rate the impact of COVID-19 on the following dimensions regarding their child, (a) sleep duration on weekdays, (b) sleep duration on weekends, (c) time spent learning at home, (d) time spent playing actively/vigorously on weekdays, (e) time spent playing actively/vigorously on weekends, (f) recreational screen time on weekdays, and (g) recreational screen time on weekends. The question layout was a matrix with four response options for each dimension regarding the impact of COVID-19. The response options included “decreased in comparison to pre-COVID period” (1), “stayed the same in comparison to pre-COVID period” (2), “increased in comparison to pre-COVID period” (3), and “I don’t know” (4). Responses of “I don’t know” were treated as system missing for the purposes of this study. Looking at the response percentages, parents reported that during the COVID-19 period, sleep time remained largely unchanged, with a small proportion reporting an increase (14.7% weekdays; 14.8% weekends) and few reporting a decrease (1.9% weekdays; 1.2% weekends), learning time mostly increased (52.2%) and rarely decreased (2.6%), while active play decreased on weekdays (38.1%) and weekends (30.9%) and less often increased (10.7% weekdays; 11.2% weekends). Screen time increased on weekdays (42.4%) and weekends (40.0%) and rarely decreased (3.1% weekdays; 2.7% weekends). For the purposes of this study, each variable was treated as a three-category outcome reflecting negative impact, no change compared to the pre-COVID period, or positive impact. Negative impact would mean perceived decreased sleep time, decreased active play, and increased time spent in SB, namely recreational screen time and time spent learning at home. In the context of movement behaviors, increased time spent learning was considered a negative impact because it involves prolonged sitting and minimal physical activity.

#### Time spent in PA, sleep, and SB

In the regular COSI Family form, parents were asked about their children’s actual post-lockdown levels of sleep, PA and SB at the time of data collection. The data collection period was considered a “post-lockdown period”, considering that less than 0.01% of parents reported that during March and April 2022, during which the data collection occurred, their child was at home due to the COVID-19 restrictions. Parents were asked “When does your child usually wake up/go to bed on school days” on weekdays, including when the child has school classes in the morning and in the afternoon. Based on this data, the typical average sleep duration was calculated. In another question, the parents were asked “Outside school hours, how much time on average per day does your child spend on playing actively at a moderate-vigorous intensity (e.g. running, jumping, playing non-supervised sports/dance, or physically active games)?“, both on weekdays and weekends. In a separate question, the parents were asked “Outside school hours, how much time on average per day does your child do homework or read a book, either at home or somewhere else?“, on both weekdays and weekends. Based on this data, the average daily time spent learning has been calculated. The average number of hours and minutes per day was calculated by weighting weekday hours (5/7) and weekend hours (2/7). Additionally, the parents were asked “Outside school hours, how much time does your child on average per day watch TV or play with electronic devices (e.g. computer, tablet, smartphone; not including moving or fitness games)?“, on weekdays and on weekends.

### Statistical analysis

The first aim of the study (risk and protective factors associated with the parental perception of COVID-19 negative impact on PA, and SB) was analyzed using multinomial logistic regression for each COVID-19 impact question. An ordinal logistic regression could not be applied due to violations of the proportional odds assumption and sparse data in certain cross-classified cells. Therefore, multinomial logistic regression models were employed as a more flexible alternative, allowing the multi-category outcome to be modeled without imposing the proportional odds constraint. Differences between categories were assessed using parameter estimates from the regression model, with one category serving as the reference group. No additional post hoc pairwise comparisons were conducted. For each COVID-19 impact question, responses were coded as “decrease”, “no change” (reference category), or “increase” compared to the pre-COVID period. Multinomial regression was not conducted for sleep time due to the very low number of responses in the “decreased” category, which made model estimation unreliable. Risk and protective factors were gender, age, weight status, and SES variables (parental education, parental employment, and perceived financial situation), and degree of urbanization. Reference categories were female, living with obesity, high SES (high parental education, no unemployed parents, no financial problems), and living in a city. To answer the second research question (parental perception of COVID-19 impact items as predictors of actual post-lockdown levels of PA, sleep, and SB), the association between parental perception of COVID-19 impact and actual post-lockdown levels of PA, sleep, and SB was analyzed using multiple linear regression analyses, with other variables (gender, age, weight status, SES variables, and degree of urbanization) used as covariates. Multicollinearity among independent variables was evaluated using variance inflation factors (VIF) in a standard linear regression model with the same predictors. All VIF values were below 3, indicating no evidence of problematic multicollinearity. To account for clustering of participants within schools and the complex survey design, multinomial logistic and linear regression models were estimated using the Complex Samples module, incorporating sampling weights and school-level clustering. Analyses were carried out using IBM SPSS 25.0.

## Results

Prevalence rates for the studied variables were calculated, and differences between groups by geographical region were tested using *Pearson *^*2*^ Test of Independence (Table 1). There were subtle differences in the sample between children from different Croatian regions. Most notably, the City of Zagreb showed the highest proportion of children with normal weight status and the lowest obesity rate, and it also had the highest share of children with highly educated and employed parents and parents who highly perceive their wealth.

**Table 1:** Distribution of sample of children in the study (%), with comparison by ^*2*^ Test of Independence

	City of Zagreb	Adriatic region	Northern region	Pannonian region	Total	*p*-value
Total (*n*)	1138	1787	1129	1383	5438	
Gender						0.587
Male	50.3	50.8	51.9	52.8	51.4	
Female	49.7	49.2	48.1	47.2	48.6	
Weight status						< 0.001
Thinness	1.4	1.1	1.7	2.3	1.6	
Normal weight	69.0	60.7	57.5	57.7	61.0	
Overweight	19.1	22.1	23.1	20.8	21.4	
Obesity	10.4	16.0	17.8	19.3	16.0	
Parental education						< 0.001
Low	27.9	43.3	55.7	61.9	47.3	
Medium	26.9	30.0	28.3	22.2	27.0	
High	45.3	26.7	16.0	16.0	25.6	
Parental employment						< 0.001
Low	22.2	32.5	25.5	38.8	30.5	
High	77.8	67.5	74.5	61.2	69.5	
Family-perceived wealth						< 0.001
Low	7.6	8.2	9.4	11.3	9.1	
Medium	40.1	42.8	43.4	45.5	43.1	
High	52.3	49.0	47.2	43.2	47.8	
Place of residence						< 0.001
Urban	100	71.5	43.9	61.6	69.1	
Rural	0.0	28.5	56.1	38.4	30.9	

Median, Q1 percentile and Q3 percentile for the continuous variables (age and actual post-lockdown behavioral levels) were calculated, and differences across geographical regions were tested using the Kruskal-Wallis nonparametric test (Table 2). There were some differences between children from different regions. Most notably, the sample in the Northern and Pannonian region had more screen time than the other two regions, both on weekdays and weekends, and the sample in the City of Zagreb, on average, spent less time sleeping and less time learning at home than other regions. The sample in the Adriatic region spent more time in active play on weekends than in other regions.

**Table 2:** Descriptive statistics of continuous variables (median, Q1 and Q3) with comparison by region using Kruskal-Wallis

		Median (Q1, Q3)				
	City of Zagreb	Adriatic region	Northern region	Pannonian region	Total sample	*p*-value
Total (n)	1138	1787	1129	1383	5438	
Age (years)	9.02 (8.48, 9.56)	9.02 (8.49, 9.51)	9.07 (8.56, 9.54)	9.06 (8.54, 9.52)	9.04 (8.51, 9.53)	0.022
Active play, weekdays (h)	1.81 (1.00, 2.00)	2.00 (1.00, 2.50)	1.50 (1.00, 2.00)	2.00 (1.00, 2.50)	2.00 (1.00, 2.50)	< 0.001
Active play, weekends (h)	3.00 (2.00, 4.00)	4.00 (2.50, 5.00)	3.00 (2.00, 4.00)	3.00 (2.00, 5.00)	3.00 (2.00, 4.50)	< 0.001
Screen time, weekdays (h)	1.00 (1.00, 2.00)	1.00 (1.00, 2.00)	1.50 (1.00, 2.00)	1.50 (1.00, 2.00)	1.00 (1.00, 2.00)	< 0.001
Screen time, weekends (h)	2.00 (2.00, 3.00)	2.00 (1.50, 3.00)	2.50 (2.00, 3.00)	2.50 (2.00, 3.00)	2.00 (2.00, 3.00)	< 0.001
Time spent learning (h)	1.28 (1.00, 1.93)	1.57 (1.00, 2.00)	1.72 (1.22, 2.14)	1.72 (1.10, 2.00)	1.57 (1.00, 2.00)	< 0.001
Sleep duration (h)	9.75 (9.50, 10.10)	10.00 (9.50, 10.25)	9.88 (9.50, 10.25)	10.00 (9.50, 10.25)	9.92 (9.50, 10.25)	< 0.001

**Fig. 1:**
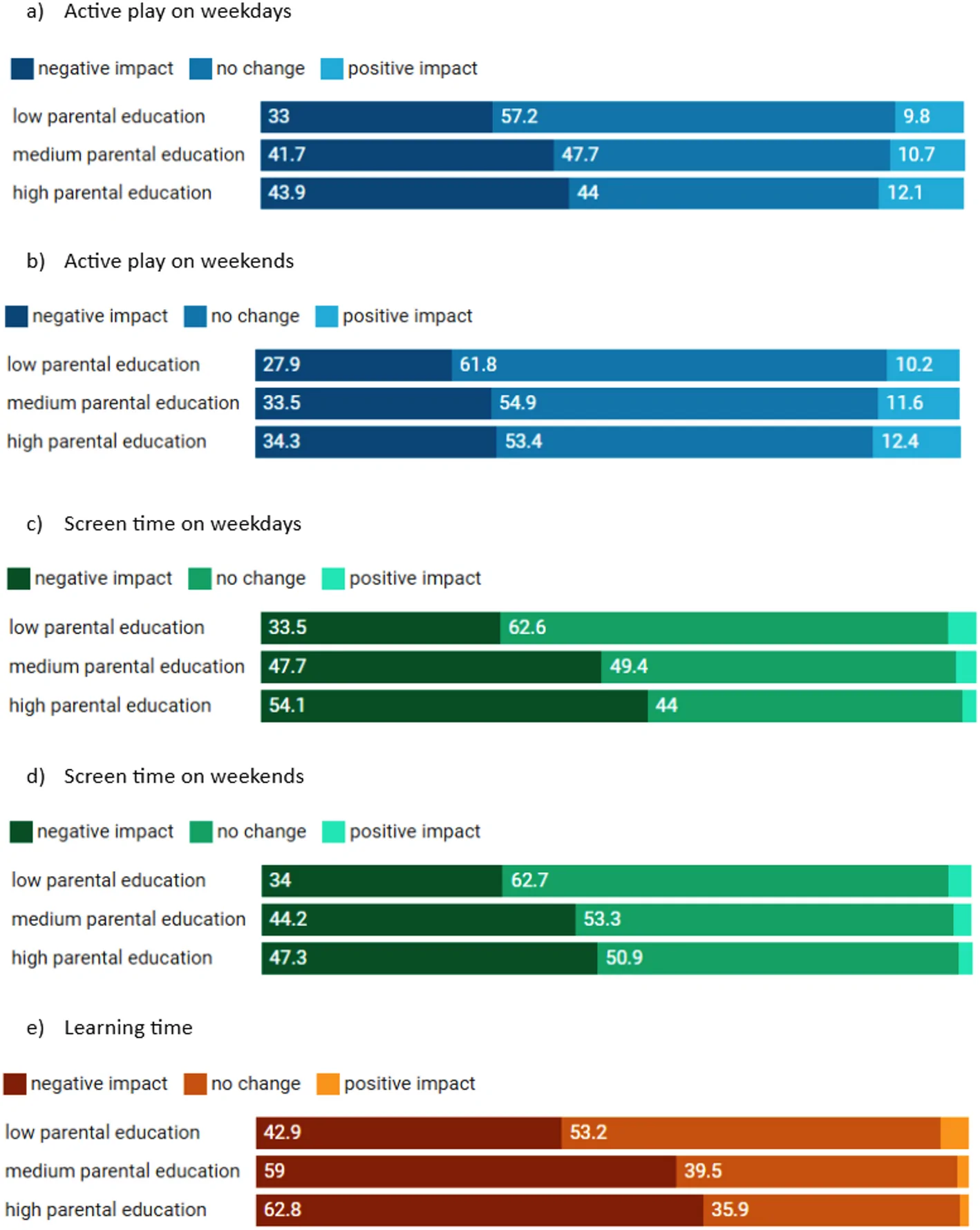
Bar chart showing the prevalence of perceived negative COVID-19 impact stratified by parental education level. Negative impact was defined as decreased active play, increased screen time and increased learning time in comparison to pre-COVID period

Bar charts were also created to illustrate the prevalence of perceived negative COVID-19 impacts stratified by parental education level, as shown below in Fig. 1.

### Risk and protective factors associated with the parental perception of COVID-19 impact on active play, screen time, and learning time

The first aim of the study was to analyze risk and protective factors associated with the parental perception of COVID-19 impact on PA and SB. Multinomial logistic regression was conducted for each COVID-19 impact question. Adjusted odds ratios (*OR*), which represent unstandardized coefficients, and their 95% confidence intervals (CI) were estimated. Results are shown in Tables 3, 4, 5, 6 and 7. Supplementary Figures S1-S3 present forest plots showing adjusted odds ratios (ORs) and 95% confidence intervals for the main predictors across the three movement behaviours (see Supplementary Information).

**Table 3:** Results of multinomial logistic regression for parental perception of COVID-19 negative impact on active play on weekdays, associated with various risk and protective factors

	95% Confidence Interval		
	OR	Lower	Upper
Age (continuous)	1.09	0.98	1.21
Gender (reference = female)			
Male	0.88*	**0.78**	**0.99**
Weight status (reference = normal weight)			
Thinness	1.17	0.72	1.90
Overweight	1.06	0.91	1.23
Obesity	1.25*	**1.04**	**1.50**
Parental education (reference = high)			
Low	0.57*	**0.48**	**0.68**
Medium	0.85	0.71	1.03
Parental employment (reference = high)			
Low	0.86*	**0.74**	**0.99**
Family-perceived wealth (reference = high)			
Low	1.40*	**1.08**	**1.81**
Medium	1.21*	**1.06**	**1.39**
Place of residence (reference = urban)			
Rural	0.81*	**0.70**	**0.94**

Bold values indicate significant confidence intervals

The reference category was “no change” while negative impact was defined as decreased active play in comparison to pre-COVID period; *Nagelkerke R*^*2*^ = 0.028; *Wald F* = 4.802; df = 22, 487; *p* < 0.001 *statistically significant (95% confidence interval does not include 1)

**Table 4:** Results of multinomial logistic regression for parental perception of COVID-19 negative impact on active play on weekends, associated with various risk and protective factors

	95% Confidence Interval		
	OR	Lower	Upper
Age (continuous)	1.17*	**1.05**	**1.31**
Gender (reference = female)			
Male	0.86*	**0.76**	**0.97**
Weight status (reference = normal weight)			
Thinness	1.02	0.61	1.70
Overweight	1.03	0.88	1.21
Obesity	1.24*	**1.03**	**1.50**
Parental education (reference = high)			
Low	0.71*	**0.59**	**0.86**
Medium	0.95	0.79	1.15
Parental employment (reference = high)			
Low	0.98	0.84	1.13
Family-perceived wealth (reference = high)			
Low	1.42*	**1.10**	**1.82**
Medium	1.17*	**1.01**	**1.35**
Place of residence (reference = urban)			
Rural	0.80*	**0.68**	**0.94**

Bold values indicate significant confidence intervals

The reference category was “no change” while negative impact was defined as decreased active play in comparison to pre-COVID period; *Nagelkerke R*^*2*^ = 0.019, *Wald F* = 3.078; df = 22, 487; *p* < 0.001 *statistically significant (95% confidence interval does not include 1).

Regarding the risk and protective factors associated with a parental perception of COVID-19 negative impact on active play compared to the “no change” category, a similar pattern was found for weekdays and weekends (Tables 3 and 4). The likelihood of parental perception of COVID-19 negative impact on active play was higher for girls than for boys (weekdays: *OR* = 0.88; CI = 0.78, 0.99; weekend: *OR* = 0.86; CI = 0.76, 0.97). The likelihood of parental perception of COVID-19 negative impact on active play was higher for children of parents with high education compared to low (weekdays: *OR* = 0.57; CI = 0.48, 0.68; weekend: *OR* = 0.71; CI = 0.59, 0.86). Children from families with low perceived wealth had 40–42% higher odds of their parents perceiving a negative COVID-19 impact on their active play compared to children from families with high perceived wealth (weekdays: *OR* = 1.40, CI = 1.08, 1.81; weekend: *OR* = 1.42, CI = 1.10, 1.82) and families with medium perceived wealth had 17–21% higher odds of their parents perceiving a negative COVID-19 impact on their active play levels compared to children from families with high perceived wealth (weekdays: *OR* = 1.21; CI = 1.06, 1.39; weekend: *OR* = 1.17; CI = 1.01, 1.35). Children with obesity had 24–25% higher odds of their parents perceiving a negative COVID-19 impact on their active play levels compared to children with normal weight status (weekdays: *OR* = 1.25; CI = 1.04, 1.50; weekend: *OR* = 1.24; CI = 1.03, 1.50). Children living in urban compared to rural places of residence had higher odds of their parents perceiving a negative COVID-19 impact on their active play (weekdays: *OR* = 0.81; CI = 0.70, 0.94; weekend: *OR* = 0.80; CI = 0.68, 0.94). Additionally, on weekends, older children had 17% higher odds of their parents perceiving a negative COVID-19 impact on their active play levels compared to younger children (*OR* = 1.17; CI = 1.05, 1.31).

In other words, female gender, living with obesity, higher parental education, lower family-perceived wealth, and living in an urban region increased the odds of parental perception of COVID-19 negative impact on active play on weekdays and weekend, while older age additionally increased the odds during weekend. Interestingly, likelihood of parental perception of COVID-19 positive impact on active play was also higher for children of parents with high education compared to low (weekdays: *OR* = 0.65; CI = 0.51, 0.84; weekend: *OR* = 0.74; CI = 0.57, 0.95), and higher for children living in urban compared to rural areas, but only on weekdays (*OR* = 0.76; CI = 0.61, 0.95). Results regarding parental perception of COVID-19 positive impact on active play are shown in Tables S1 and S2 (see Supplementary Information).

**Table 5:** Results of multinomial logistic regression for parental perception of COVID-19 negative impact on screen time on weekdays, associated with various risk and protective factors

	95% Confidence Interval		
	OR	Lower	Upper
Age (continuous)	1.22*	**1.10**	**1.36**
Gender (reference = female)			
Male	1.10	0.98	1.25
Weight status (reference = normal weight)			
Thinness	0.81	0.49	1.34
Overweight	0.96	0.82	1.13
Obesity	1.30*	**1.10**	**1.53**
Parental education (reference = high)			
Low	0.48*	**0.40**	**0.56**
Medium	0.83*	**0.70**	**0.98**
Parental employment (reference = high)			
Low	0.76*	**0.66**	**0.88**
Family-perceived wealth (reference = high)			
Low	1.36*	**1.07**	**1.74**
Medium	1.09	0.96	1.23
Place of residence (reference = urban)			
Rural	0.73*	**0.62**	**0.84**

Bold values indicate significant confidence intervals

The reference category was “no change” while negative impact was defined as increased screen time in comparison to pre-COVID period; *Nagelkerke R*^*2*^ = 0.062, *Wald F* = 9.791; df = 22, 487; *p* < 0.001 *statistically significant (95% confidence interval does not include 1)

**Table 6:** Results of multinomial logistic regression for parental perception of COVID-19 negative impact on screen time on weekend, associated with various risk and protective factors

	95% Confidence Interval		
	OR	Lower	Upper
Age (continuous)	1.19*	**1.06**	**1.32**
Gender (reference = female)			
Male	1.15*	**1.02**	**1.29**
Weight status (reference = normal weight)			
Thinness	0.93	0.58	1.51
Overweight	1.05	0.90	1.23
Obesity	1.37*	**1.16**	**1.62**
Parental education (reference = high)			
Low	0.59*	**0.50**	**0.70**
Medium	0.90	0.76	1.07
Parental employment (reference = high)			
Low	0.83*	**0.72**	**0.95**
Family-perceived wealth (reference = high)			
Low	1.51*	**1.19**	**1.91**
Medium	1.16*	**1.02**	**1.32**
Place of residence (reference = urban)			
Rural	0.75*	**0.65**	**0.88**

Bold values indicate significant confidence intervals

The reference category was “no change” while negative impact was defined as increased screen time in comparison to pre-COVID period; *Nagelkerke R*^*2*^ = 0.041, *Wald F* = 6.278; df = 22, 487; *p* < 0.001 *statistically significant (95% confidence interval does not include 1)

Regarding the factors associated with a parental perception of COVID-19 negative impact on screen time, a similar pattern was found for weekdays and weekends (Tables 5 and 6). Older children had 19–22% higher odds of their parents perceiving a negative COVID-19 impact on their screen time compared to younger children (weekdays: *OR* = 1.22; CI = 1.07, 1.39; weekend: *OR* = 1.19; CI = 1.06, 1.32). Children with obesity had 30–37% higher odds of their parents perceiving a negative COVID-19 impact on their screen time than children with normal weight status (weekdays: *OR* = 1.30, CI = 1.10, 1.53; weekend: *OR* = 1.37, CI = 1.16, 1.62). Furthermore, the likelihood of parental perception of COVID-19 negative impact on screen time was higher for children of parents with high education compared to low (weekdays: *OR* = 0.48; CI = 0.40, 0.56; weekend: *OR* = 0.59; CI = 0.50, 0.70), for children of parents with high compared to low employment (weekdays: *OR* = 0.76; CI = 0.66, 0.88; weekend: *OR* = 0.83; CI = 0.72, 0.95). Children from families with low perceived wealth had 36–51% higher odds of their parents perceiving a negative COVID-19 impact on their screen time compared to children from families with high perceived wealth (weekdays: *OR* = 1.36; CI = 1.07, 1.74; weekend: *OR* = 1.51; CI = 1.19, 1.91). The likelihood of parental perception of COVID-19 negative impact on screen time was also higher for children living in urban compared to rural places (weekdays: *OR* = 0.73; CI = 0.62, 0.84; weekend: OR = 0.75; CI = 0.65, 0.88). Additionally, on weekends, boys had 15% higher odds of their parents perceiving a negative COVID-19 impact on their screen time than girls (*OR* = 1.15; CI = 1.02, 1.29). In other words, higher child age, high parental education, no unemployed parents, living with obesity, lower family-perceived wealth, and living in a city increased the odds of parental perception of COVID-19 negative impact on screen time on weekdays and weekend, while male gender additionally increased the odds during weekend.

While having both employed parents increased the odds of a parental perception of COVID-19 negative impact on screen time, on weekdays it also decreased the likelihood of a parental perception of COVID-19 positive impact on screen time, relative to reporting no change (*OR* = 1.46; CI = 1.01, 2.10). On the other hand, while low perceived wealth increased the odds of a parental perception of COVID-19 negative impact on screen time, on weekends it also increased the likelihood of a parental perception of COVID-19 positive impact on screen time (*OR* = 2.07; CI = 1.11, 3.83). Results regarding parental perception of COVID-19 positive impact on screen time are shown in Tables S3 and S4 (see Supplementary Information).

**Table 7:** Results of multinomial logistic regression parental perception of COVID-19 negative impact on learning time associated with various risk and protective factors

	95% Confidence Interval		
	OR	Lower	Upper
Age (continuous)	1.30*	**1.15**	**1.46**
Gender (reference = female)			
Male	0.94	0.84	1.07
Weight status (reference = normal weight)			
Thinness	0.80	0.49	1.29
Overweight	1.01	0.87	1.18
Obesity	0.95	0.80	1.13
Parental education (reference = high)			
Low	0.49*	**0.41**	**0.58**
Medium	0.85	0.72	1.01
Parental employment (reference = high)			
Low	0.77*	**0.74**	**0.89**
Family-perceived wealth (reference = high)			
Low	1.21	0.93	1.58
Medium	1.16*	**1.02**	**1.32**
Place of residence (reference = urban)			
Rural	0.93	0.79	1.09

Bold values indicate significant confidence intervals

The reference category was “no change” while negative impact was defined as increased learning time in comparison to pre-COVID period; Nagelkerke *R*^*2*^ = 0.059, *Wald F* = 8.244; df = 22, 486; *p* < 0.001 *statistically significant (95% confidence interval does not include 1)

Older children had 30% higher odds of their parents perceiving a negative COVID-19 impact on learning time compared to younger children (*OR* = 1.30; CI = 1.15, 1.46) (Table 7). Furthermore, the likelihood was higher for children of parents with high education compared to low (*OR* = 0.49; CI = 0.41, 0.58), and for children of parents with high compared to low employment (*OR* = 0.77; CI = 0.67, 0.89).

In other words, higher child age, high parental education and high parental employment increased the odds of a parental perception of COVID-19 negative impact on time spent learning at home (i.e., an increase in this sedentary behavior).

Interestingly, the likelihood of a decrease in learning time (i.e., a parental perception of COVID-19 positive impact in the context of movement behavior) also increased with children’s age (*OR* = 1.67; CI = 1.22, 2.28). Furthermore, children of parents with lower employment were more likely to have a parental perception of COVID-19 positive impact regarding learning time (*OR* = 1.78; CI = 1.19, 2.66). Results regarding a parental perception of COVID-19 positive impact on learning time are shown in Table S5 (see Supplementary Information).

### Association between parental perception of COVID-19 impact items with actual post-lockdown behavioral levels of PA, sleep and SB

The second aim of the study was to analyze the association between parental perception of COVID-19 impact items and actual post-lockdown levels of PA, sleep and SB, while controlling for child age, gender, weight status, parental education, parental employment, perceived family wealth, and place of residence. Multiple complex samples general linear models were conducted. Parental perception of COVID-19 impact items were treated as predictors reflecting perceived changes in children’s behaviors coded as decreased, stayed the same (reference category), or increased compared to the pre-COVID period. The results show that children whose parents perceived a decrease in active play during the COVID-19 period had lower post-lockdown levels of playtime on both weekdays and weekends compared to those who reported no change (weekdays: *B* = − 0.20; CI = − 0.27, − 0.14; weekends: *B* = − 0.20; CI = − 0.31, − 0.08). In practical terms, a parental perception of perceived negative COVID-19 impact on active play was associated with approximately 12 min less active play per weekday and 12 min less per weekend day, amounting to an estimated 1.4 h less active play per week. Conversely, a parental perception of increase in active play was associated with higher actual post-lockdown levels of weekend playtime (*B* = 0.34; CI = 0.19, 0.49) but showed no significant difference on weekdays. Furthermore, a parental perception of increase in screen time was associated with higher post-lockdown screen time on both weekdays and weekends (weekdays: *B* = 0.20; CI = 0.15, 0.25; weekends: *B* = 0.46; CI = 0.39, 0.54). In practical terms, a perceived increase in screen time was associated with approximately 12 min more per weekday and 28 min more per weekend day, corresponding to nearly 1.9 h more screen time per week post-lockdown. Conversely, children whose parents perceived a decrease in screen time during the COVID-19 period had lower post-lockdown screen time on both weekdays and weekends compared to those who reported no change (weekdays: *B* = − 0.18; CI = − 0.33, − 0.03; weekends: *B* = − 0.33; CI = − 0.56, − 0.10). A parental perception of perceived increase in learning time during the COVID-19 period was not significantly associated with post-lockdown learning time, and perceived changes in sleep during the COVID-19 period were also not associated with post-lockdown sleep duration. Results are shown in Tables S6-S11 (see Supplementary Information).

## Discussion

Findings of this nationally representative study conducted on Croatian school-aged children provide important insights into how the COVID-19 pandemic and related restrictions have shaped children’s movement behaviors. Previously published results of the CroCOSI 2021/2022 study [43], along with international evidence, suggests that the pandemic may have had a measurable negative impact on children’s PA and SB [25, 26, 29]. We aimed to examine whether the parental perception of COVID-19 negative impact in the CroCOSI study was associated with specific child- and family-level variables that served as risk or protective factors. In other words, in the first aim of this cross-sectional study we employed multinomial logistic regression to analyze the likelihood of parental perception of COVID-19 negative impact, no change, or positive impact on children’s movement behaviors, while accounting for specific child demographic and anthropometric characteristics, and the family’s socioeconomic background.

### Correlates of perceived impact of COVID-19 on children’s movement behavior

Our findings indeed suggest that the COVID-19 pandemic may not have affected all children and their movement behavior equally, and that some family and child characteristics served as risk or protective factors regarding the time spent in PA, SB. Family socioeconomic characteristics, for instance, served as significant determinants of the parental perception of COVID-19 negative impact on children’s movement behavior. High parental education, in particular, was consistently associated with a parental perception of negative impact of COVID-19 across all examined movement behaviors - active play, and SB (recreational screen time and time spent learning at home). This finding should be interpreted with caution, as it may reflect both actual behavioral changes and differences in parental perception. Higher educated parents may have greater awareness of movement behavior guidelines and may be more sensitive to deviations from pre-pandemic routines [e.g. 50] particularly given their greater reliance on structured activities such as organized sports [e.g. 25, 51]. Consequently, even relatively small reductions in activity or increases in screen time may be perceived as meaningful. At the same time, these families may have experienced greater disruption of structured opportunities for physical activity during the pandemic [37], which could result in genuine behavioral changes and increased sedentary behavior. Therefore, the observed association likely reflects a combination of real changes and heightened parental awareness, highlighting the importance of considering reporting bias when interpreting COVID-related behavioral change measures. Interestingly, in our study, high parental education was also associated with an increased likelihood of parental perception of COVID-19 positive impact on active play, suggesting that children of parents with higher education were more likely to experience any changes in their active play during COVID-19 pandemic (also possibly reflecting greater sensitivity to deviations from pre-pandemic routines), whereas parents with lower education tended to perceive more stable playtime. Evidence from other countries show mixed results regarding the relationship between parental education and children’s movement behavior. Consistent with our findings, a Chilean study found that, among young children, higher parental education was associated with greater increases in screen time and greater decreases in PA behaviors during early stages of the COVID-19 pandemic [52]. A larger increase of sedentary lifestyle among children with at least one parent with a medium or high educational level was also found in the Italian study conducted by Nardone et al. [37], which was also conducted as part of the international COSI study. On the other hand, Spanish children whose mothers had non-university education showed a greater decrease in PA during the confinement compared to their peers whose mothers had university education [53]. Accordingly, a study conducted in Switzerland found that, two years into the COVID-19 pandemic, the odds of decreased PA were higher among children of parents with a lower educational level [36]. However, it is essential to consider the way PA was operationalized across studies. While the studies referenced above measured overall PA in children, our study specifically examined active play of moderate to vigorous intensity, such as running, jumping, and indoor fitness activities.

In our study, children from families with low family-perceived wealth were more likely to have a parental perception of COVID-19 negative impact on active play and screen time during the COVID-19 pandemic, while on weekends lower perceived wealth was also linked to a higher likelihood of parental perception of COVID-19 positive impact on screen time. These findings, considering the findings regarding parental education, highlight the complex relationship between different aspects of socioeconomic status and the parental perception of COVID-19 impact on children’s movement behaviors. The same pattern was found in the comparable Italian study, conducted on the sample of children enrolled in the third grade of primary school [37]. In line with our results, Seguin et al. [54] found that lower household income was associated with increased screen time hours during the pandemic, and McArthur et al. [55] found that children’s recreational screen time during the pandemic was most elevated in households with high financial and psychological stress. Average-to-poor financial circumstances were associated with increased recreational screen time in children from Switzerland [36], as well as with both increases and decreases in PA. Children from disadvantaged households were overall more likely to experience changes in health behaviors, both positive and negative [36]. In a study conducted in Germany, children from low-income families also showed greater reduction in physical activity [42]. On the contrary, a Canadian study reported significant declines in PA during the COVID-19 pandemic among children from upper- and upper-middle-income families (assessed using a neighborhood-level household income measure), whereas children from low- and middle-income families did not show significant changes [56]. Again, it should be emphasized that in their study, PA was defined as any activity that makes the child out of breath or increases body temperature, which also includes organized activities, such as sports clubs or other structured activities, often requiring parental fees, which may account for the more significant changes observed in PA among higher-income families in their study.

Furthermore, living in an urban area, compared to a rural area, was associated with a parental perception of COVID-19 negative impact on screen time, and active play. The urban living environment has been identified in numerous studies as a risk factor for a greater negative impact of the COVID-19 pandemic on children’s movement behavior. Living in an urban environment was significantly associated with decreased PA and increased screen time among French children [57]. Several other European studies found that having access to a garden/yard was a protective factor in the context of children’s PA during the lockdown [37–39]. Having access to a garden/yard provided the opportunity for an outdoor play during the pandemic, which could have also led to a less need to engage in screen time [38]. Francisco et al. [58] found that having an outdoor exit (e.g., a garden or terrace) was associated with lower levels of psychological and behavioral symptomatology in children during the pandemic. In urban environments, it was likely more difficult to use outdoor spaces to provide opportunities for active play while also adhering to COVID-19 restrictions. Safe neighborhood outdoor areas, such as local parks, private gardens, meadows, and back/front yards, were essential settings for children to remain physically active throughout the day [31, 59]. Interestingly, on weekdays, living in an urban area also increased the likelihood of a parental perception of COVID-19 positive impact on active play, suggesting that children living in urban areas were more likely to experience any changes in active play during weekdays, while children from rural areas maintained more stable levels of active play with fewer changes throughout the week.

Our study findings also indicate that, according to parents, children from families with high parental employment were more likely to have a parental perception of COVID-19 negative impact on SB (screen time and time spent learning at home). In cases where both parents were employed and the child spent time at home due to lockdown measures, it might have been more difficult to control whether and for what purpose the child used screen-based devices. Our results are in line with those from Seguin et al. [54], where the lack of parental involvement was associated with increased hours of screen usage in children during the initial months of the pandemic. Furthermore, interviews with parents in the study by Riazi et al. [60] showed that an increase in screen time was often associated with pandemic-related factors such as the shift to online learning, limited opportunities for outdoor activities, increased need for virtual socialization, and the use of screen-based devices as a form of childcare when parents were working, including working from home. In our study, low parental employment served as a protective factor for the perceived COVID-19 impact on children’s screen time and time spent learning (SB).

Regarding child-level characteristics, older age was significantly associated with the parental perception of COVID-19 negative impact on SB, and active play during weekends, consistent with other studies. For example, in the study conducted in the United States parents of older children (ages 9–13) vs. younger children (ages 5–8) reported greater decreases in PA and greater increases in SB during the pre- to early-COVID-19 period [30]. Some other studies found that, on average, younger children experienced less change in their pre-COVID-19 movement behaviors than older children [36, 39]. Although it was found that PA declines and SB increases as children get older [61–63], the COVID-19 pandemic may be accelerating these changes [30, 55]. Our findings also show that, according to parental reports, female children were more likely than male children to have a parental perception of COVID-19 negative impact on active play during both weekdays and weekends. In line with our findings, Ostermeier et al. [56] found that girls experienced a larger reduction in PA per week during COVID-19 pandemic than boys. On the other hand, parents reported that male children had a higher likelihood of parental perception of COVID-19 negative impact on screen time, but only on weekends. In Schmidt et al.‘s study [64], boys showed a greater increase in recreational screen time than girls among 6-10-year-olds, but not among 11-17-year-olds. Medrano et al. [53] reported that boys increased their screen time more than girls during the pandemic, and the study by Ostermeier et al. [56] found that recreational screen time significantly increased for boys and girls during COVID-19, with boys showing a greater increase in weekday screen time than girls. Finally, parents of children with obesity were more likely to report a parental perception of COVID-19 negative impact on active play and screen time compared with parents of children with normal weight, suggesting that children with obesity may have been particularly vulnerable to pandemic-related changes in daily movement routines.

### Perceived negative COVID-19 impact as predictor of post-lockdown movement behavior

As part of the second research question, which aimed to explore the association between the parental perception of COVID-19 impact and actual post-lockdown PA, sleep and SB levels, our study found that the parental perception of COVID-19 negative impact on children’s movement behavior was associated with actual post-lockdown behavioral levels of active play and screen time. In practical terms, a parental perception of COVID-19 negative impact on active play was associated with approximately 12 min less active play per weekday and 12 min less per weekend day post-lockdown, adding up to nearly 1.4 h less active play per week. Similarly, a perceived increase in screen time during COVID-19 was associated with approximately 12 min more screen time per weekday and 28 min more screen time per weekend day post-lockdown, corresponding to nearly 1.9 h more screen time per week. The relationship between the parental perception of COVID-19 negative impact and actual post-lockdown behavioral levels highlights the potential for prolonged lifestyle changes in children beyond the acute phases of the COVID-19 pandemic. Reduced opportunities for spontaneous active play and outdoor activities during lockdown, combined with elevated screen time for educational purposes and socializing, may have established new behavioral norms for children. Although all children experienced some level of disruption, differences by socioeconomic background and demographic factors suggest that family and children’s characteristics mediated the extent of behavioral change.

### Limitations and strengths

Several limitations of this study should be noted. Firstly, the study relied on parental reports regarding changes in children’s movement behavior during the COVID-19 pandemic, which could be subject to recall or social desirability bias. Perceived impact may also reflect parental awareness rather than objective behavioral change. The absence of pre-pandemic behavioral data prevents the quantification of actual behavioral change, meaning that the findings reflect perceived shifts rather than measured transitions. Furthermore, the reliance on parental reports without objective measures of physical activity, such as accelerometry, limits the precision of the data. Additionally, socioeconomic variables such as parental employment status or family income may have changed during the pandemic, leaving our measures unable to capture families’ circumstances at that time fully. An important finding of the present study is the divergent pattern observed for parental education and family-perceived wealth. While higher parental education was associated with greater likelihood of reporting negative COVID-19-related changes in children’s movement behaviors, lower perceived wealth was also associated with a perceived negative impact. This apparent discrepancy suggests that these socioeconomic indicators capture distinct underlying mechanisms. Parental education may reflect greater health literacy, awareness of behavioral guidelines, and higher expectations regarding children’s routines, leading to increased sensitivity to behavioral changes. In contrast, perceived family wealth is more closely related to material resources and environmental constraints, such as access to space, equipment, and safe outdoor environments, which may have directly limited children’s opportunities for physical activity during the pandemic. Therefore, while lower wealth likely reflects genuine behavioral constraints, higher parental education may be associated with heightened perception and reporting of change. These findings highlight the importance of distinguishing between different dimensions of socioeconomic status when interpreting parent-reported measures of behavioral change. Also, as mentioned, a relatively small proportion of parents in our study reported a negative impact of COVID-19 on their child’s sleep, which is why a multinomial regression could not be conducted to examine risk and protective factors associated with the perceived COVID-19 impact on sleep. COSI-COVID report [25] also suggests that the sleep behaviors remained the same for most children during the pandemic. Furthermore, while the cross-sectional study design captures associations between the perceived COVID-19 impact and post-lockdown behaviors, causal inferences could not be made, and the potential for reverse causality must be considered. Given the number of regression models and predictors examined, multiple testing may have increased the risk of Type I error, and the findings should therefore be interpreted as exploratory. Finally, the findings are based on a national sample, which may limit their generalizability to populations beyond the Croatian context. Nevertheless, the study’s strengths include its large, nationally representative sample and the use of standardized COSI methodology, both of which enhance the generalizability of the findings. Furthermore, a distinct contribution of this research lies in its comprehensive exploration of potential correlates regarding parental perceptions of the COVID-19 impact on children’s movement behavior.

## Conclusions

This study explored the parental perceptions of negative COVID-19 impact on children’s movement behavior (reductions in active play and sleep, and increases in screen time and time spent learning at home) in Croatian school-aged children, aiming to examine specific child- and family-related variables that posed a greater risk for experiencing the negative COVID-19 impact. Our findings indicate that the parental perception of negative COVID-19 impact on children’s movement behavior was strongly patterned by socioeconomic factors, child-related characteristics such as age and weight status, and degree of urbanization. Regarding the second study aim, the results showed that the parental perception of the negative COVID-19 impact on children’s movement behavior was associated with children’s post-lockdown daily routines, namely active play and screen time duration. These findings highlight the need for public health strategies that support the re-establishment of healthy activity patterns, which should prioritize equitable access to safe opportunities for active play and other forms of PA, and could help reduce excessive screen use in children. Such multisectoral, continuous measures are crucial not only for mitigating the potential effects of COVID-19 but also for addressing the ongoing challenge of childhood obesity. The unique contribution of this study is its use of a large, nationally representative sample and standardized COSI methodology, providing objectively grounded data on a wide range of correlates. To our knowledge, this is currently the only study in Croatia to have examined these specific dynamics on a national scale.

## Supplementary Information

Below is the link to the electronic supplementary material.

Supplementary Material 1.

## Data Availability

The original contributions presented in this study are available within the paper and its Supplementary Information. Further inquiries can be directed to the corresponding author.
