# Supplementary material for: Sociodemographic correlates of perceived COVID-19 impact on movement behaviors in Croatian school-aged children
Source: Discov Public Health. 2026 Oct 7;23(1):1603. doi: 10.1186/s12982-026-02827-9 (PMC13645849; doi:10.1186/s12982-026-02827-9)
Supplement: Supplementary file 1 [file 12982_2026_2827_MOESM1_ESM.docx]

**Table S1** Results of multinomial logistic regression for positive COVID-19 impact on active play on weekdays associated with various risk and protective factors

|  |  | **95% Confidence Interval** | |
| --- | --- | --- | --- |
|  | ***OR*** | **Lower** | **Upper** |
| **Age (continuous)** | 1.12 | 0.96 | 1.30 |
| **Gender** (reference = female) |  |  |  |
| Male | 1.08 | 0.88 | 1.32 |
| **Weight status** (reference = normal weight) |  |  |  |
| Thinness | 0.68 | 0.26 | 1.77 |
| Overweight | 1.09 | 0.85 | 1.39 |
| Obesity | 1.01 | 0.76 | 1.34 |
| **Parental education** (reference = high) |  |  |  |
| Low | 0.65* | **0.51** | **0.84** |
| Medium | 0.84 | 0.64 | 1.11 |
| **Parental employment** (reference = high) |  |  |  |
| Low | 0.89 | 0.71 | 1.13 |
| **Family-perceived wealth** (reference = high) |  |  |  |
| Low | 1.28 | 0.87 | 1.86 |
| Medium | 1.19 | 0.97 | 1.46 |
| **Place of residence** (reference = urban) |  |  |  |
| Rural | 0.76* | **0.61** | **0.95** |

Notes. The reference category was “no change” while positive impact was defined as increased active play in comparison to pre-COVID period; *Nagelkerke R^2^* = 0.028; *Wald* *F* = 4.802; df = 22, 487; *p* < 0.001; *statistically significant (95% confidence interval does not include 1)

**Table S2** Results of multinomial logistic regression for positive COVID-19 impact on active play on weekend associated with various risk and protective factors

|  |  | **95% Confidence Interval** | |
| --- | --- | --- | --- |
|  | ***OR*** | **Lower** | **Upper** |
| **Age (continuous)** | 1.00 | 0.85 | 1.18 |
| **Gender** (reference = female) |  |  |  |
| Male | 1.03 | 0.83 | 1.26 |
| **Weight status** (reference = normal weight) |  |  |  |
| Thinness | 0.56 | 0.22 | 1.43 |
| Overweight | 1.11 | 0.89 | 1.39 |
| Obesity | 0.82 | 0.61 | 1.10 |
| **Parental education** (reference = high) |  |  |  |
| Low | 0.74* | **0.57** | **0.95** |
| Medium | 0.93 | 0.72 | 1.21 |
| **Parental employment** (reference = high) |  |  |  |
| Low | 1.00 | 0.80 | 1.23 |
| **Family-perceived wealth** (reference = high) |  |  |  |
| Low | 1.05 | 0.71 | 1.55 |
| Medium | 1.02 | 0.83 | 1.25 |
| **Place of residence** (reference = urban) |  |  |  |
| Rural | 0.83 | 0.65 | 1.06 |

Notes. the reference category was "no change" while positive impact was defined as increased active play in comparison to pre-COVID period; *Nagelkerke R^2^* = 0.019. *F* = 3.07; df = 22, 487; *p* < 0.001; *statistically significant (95% confidence interval does not include 1)

**Table S3** Results of multinomial logistic regression for positive COVID-19 impact on screen time on weekdays associated with various risk and protective factors

|  |  | **95% Confidence Interval** | |
| --- | --- | --- | --- |
|  | **OR** | **Lower** | **Upper** |
| **Age (continuous)** | 1.09 | 0.83 | 1.43 |
| **Gender** (reference = female) |  |  |  |
| Male | 0.78 | 0.56 | 1.09 |
| **Weight status** (reference = normal weight) |  |  |  |
| Thinness | 0.34 | 0.05 | 2.53 |
| Overweight | 1.01 | 0.66 | 1.55 |
| Obesity | 1.01 | 0.60 | 1.70 |
| **Parental education** (reference = high) |  |  |  |
| Low | 1.20 | 0.74 | 1.94 |
| Medium | 1.24 | 0.71 | 2.14 |
| **Parental employment** (reference = high) |  |  |  |
| Low | 1.46* | **1.01** | **2.10** |
| **Family-perceived wealth** (reference = high) |  |  |  |
| Low | 1.61 | 0.88 | 2.93 |
| Medium | 0.98 | 0.67 | 1.43 |
| **Place of residence** (reference = urban) |  |  |  |
| Rural | 0.87 | 0.60 | 1.28 |

Notes. the reference category was "no change" while positive impact was defined as decreased screen time in comparison to pre-COVID period; *Nagelkerke R^2^* = 0.062; *F* = 9.791; df = 22, 487; *p* < 0.001; *statistically significant (95% confidence interval does not include 1)

**Table S4** Results of multinomial logistic regression for positive COVID-19 impact on screen time on weekend associated with various risk and protective factors

|  |  | **95% Confidence Interval** | |
| --- | --- | --- | --- |
|  | ***OR*** | **Lower** | **Upper** |
| **Age (continuous)** | 1.01 | 0.74 | 1.36 |
| **Gender** (reference = female) |  |  |  |
| Male | 0.77 | 0.54 | 1.11 |
| **Weight status** (reference = normal weight) |  |  |  |
| Thinness | 0.41 | 0.06 | 3.02 |
| Overweight | 0.76 | 0.46 | 1.24 |
| Obesity | 1.06 | 0.63 | 1.76 |
| **Parental education** (reference = high) |  |  |  |
| Low | 1.10 | 0.66 | 1.82 |
| Medium | 1.18 | 0.68 | 2.07 |
| **Parental employment** (reference = high) |  |  |  |
| Low | 1.33 | 0.91 | 1.95 |
| **Family-perceived wealth** (reference = high) |  |  |  |
| **Low** | 2.07* | **1.11** | **3.83** |
| Medium | 1.01 | 0.67 | 1.51 |
| **Place of residence** (reference = urban) |  |  |  |
| Rural | 0.80 | 0.53 | 1.21 |

Notes. The reference category was “no change” while positive impact was defined as decreased screen time in comparison to pre-COVID period; *Nagelkerke R^2^* = 0.041; *F* = 6.278; df = 22, 487; *p* < 0.001; *statistically significant (95% confidence interval does not include 1)

**Table S5** Results of multinomial logistic regression for positive COVID-19 impact on learning time on weekend associated with various risk and protective factors

|  |  | **95% Confidence Interval** | |
| --- | --- | --- | --- |
|  | ***OR*** | **Lower** | **Upper** |
| **Age (continuous)** | 1.67* | **1.22** | **2.28** |
| **Gender** (reference = female) |  |  |  |
| Male | 1.02 | 0.70 | 1.50 |
| **Weight status** (reference = normal weight) |  |  |  |
| Thinness | 1.35 | 0.42 | 4.35 |
| Overweight | 1.01 | 0.64 | 1.58 |
| Obesity | 0.72 | 0.41 | 1.28 |
| **Parental education** (reference = high) | 1.35 | 0.42 | 4.35 |
| Low | 1.61 | 0.88 | 2.95 |
| Medium | 0.99 | 0.49 | 2.01 |
| **Parental employment** (reference = high) |  |  |  |
| Low | 1.78* | **1.19** | **2.66** |
| **Family-perceived wealth** (reference = high) |  |  |  |
| Low | 1.37 | 0.73 | 2.35 |
| Medium | 1.23 | 0.83 | 1.83 |
| **Place of residence** (reference = urban) |  |  |  |
| Rural | 0.77 | 0.49 | 1.22 |

Notes. the reference category was "no change" while positive impact was defined as decreased learning time in comparison to pre-COVID period; *Nagelkerke R^2^ =* 0.059; *F* = 8.244; df = 22, 486; *p* < 0.001; *statistically significant (95% confidence interval does not include 1)

**Figure S1** Forest plot showing adjusted ORs and 95% confidence intervals for the main predictors. Results are derived from multinomial logistic regressions for parental perception of COVID-19 negative impact on active play on weekdays & weekends, associated with various protective factors

Notes. The reference category was "no change" while negative impact was defined as decreased active play in comparison to pre-COVID period. Reference categories for the correlates were: female, normal weight, high parental education, high parental employment, high family-perceived wealth, and urban place of residence

**Figure S2** Forest plot showing adjusted ORs and 95% confidence intervals for the main predictors. Results are derived from multinomial logistic regressions for parental perception of COVID-19 negative impact on screen time on weekdays & weekends, associated with various protective factors

Notes. The reference category was "no change" while negative impact was defined as increased screen time in comparison to pre-COVID period. Reference categories for the correlates were: female, normal weight, high parental education, high parental employment, high family-perceived wealth, and urban place of residence

**Figure S3** Forest plot showing adjusted ORs and 95% confidence intervals for the main predictors. Results are derived from multinomial logistic regressions for parental perception of COVID-19 negative impact on learning time, associated with various protective factors

Notes. The reference category was "no change" while negative impact was defined as increased learning time in comparison to pre-COVID period. Reference categories for the correlates were: female, normal weight, high parental education, high parental employment, high family-perceived wealth, and urban place of residence

**Table S6** Results of multiple linear regression examining the association between perceived COVID-19 impact on active play and post-lockdown levels of active play on weekdays

|  |  | **95% Confidence Interval** | |
| --- | --- | --- | --- |
|  | ***B*** | **Lower** | **Upper** |
| **Age (continuous)** | -0.07* | -0.12 | -0.01 |
| **Gender** (reference = female) |  |  |  |
| Male | 0.10* | 0.04 | 0.16 |
| **Weight status** (reference = normal weight) |  |  |  |
| Thinness | -0.04 | -0.21 | 0.13 |
| Overweight | -0.05 | -0.12 | 0.02 |
| Obesity | -0.18* | -0.27 | -0.09 |
| **Parental education** (reference = high) |  |  |  |
| Low | 0.35* | 0.26 | 0.43 |
| Medium | 0.09 | 0.00 | 0.17 |
| **Parental employment** (reference = high) |  |  |  |
| Low | 0.12* | 0.05 | 0.19 |
| **Family-perceived wealth** (reference = high) |  |  |  |
| Low | -0.05 | -0.18 | 0.07 |
| Medium | 0.05 | -0.01 | 0.12 |
| **Place of residence** (reference = urban) |  |  |  |
| Rural | 0.05 | -0.02 | 0.13 |
| **COVID-19 impact** (reference = no change) |  |  |  |
| Decreased | -0.20* | -0.27 | -0.14 |
| Increased | 0.01 | -0.10 | 0.11 |

Notes. *R^2^* = 0.052; *Wald F* = 17.254; df = 13, 491; *p* < .000; *statistically significant (95% confidence interval does not include 0)

**Table S7** Results of multiple linear regression examining the association between perceived COVID-19 impact on active play and post-pandemic levels of active play on weekend

|  |  | **95% Confidence Interval** | |
| --- | --- | --- | --- |
|  | ***B*** | **Lower** | **Upper** |
| **Age (continuous)** | -0.10* | -0.19 | -0.01 |
| **Gender** (reference = female) |  |  |  |
| Male | 0.21* | 0.11 | 0.31 |
| **Weight status** (reference = normal weight) |  |  |  |
| Thinness | 0.05 | -0.23 | 0.32 |
| Overweight | -0.11 | -0.23 | 0.02 |
| Obesity | -0.28* | -0.42 | -0.14 |
| **Parental education** (reference = high) |  |  |  |
| Low | 0.52* | 0.39 | 0.64 |
| Medium | 0.25* | 0.12 | 0.37 |
| **Parental employment** (reference = high) |  |  |  |
| Low | 0.12 | -0.01 | 0.24 |
| **Family-perceived wealth** (reference = high) |  |  |  |
| Low | -0.09 | -0.29 | 0.12 |
| Medium | 0.13* | 0.03 | 0.24 |
| **Place of residence** (reference = urban) |  |  |  |
| Rural | -0.01 | -0.13 | 0.12 |
| **COVID-19 impact** (reference = no change) |  |  |  |
| Decreased | -0.20* | -0.31 | -0.08 |
| Increased | 0.34* | 0.19 | 0.49 |

Notes. *R^2^* = 0.038; *Wald F* = 12.893; df = 13, 491; *p* < .001; *statistically significant (95% confidence interval does not include 0)

**Table S8** Results of multiple linear regression examining the association between perceived COVID-19 impact on screen time and post-pandemic levels of screen time on weekdays

|  |  | **95% Confidence Interval** | |
| --- | --- | --- | --- |
|  | ***B*** | **Lower** | **Upper** |
| **Age (continuous)** | 0.13* | 0.08 | 0.18 |
| **Gender** (reference = female) |  |  |  |
| Male | 0.07* | 0.04 | 0.14 |
| **Weight status** (reference = normal weight) |  |  |  |
| Thinness | 0.08 | -0.11 | 0.27 |
| Overweight | -0.02 | -0.08 | 0.05 |
| Obesity | 0.06 | -0.01 | 0.14 |
| **Parental education** (reference = high) |  |  |  |
| Low | 0.22* | 0.15 | 0.29 |
| Medium | 0.11* | 0.05 | 0.18 |
| **Parental employment** (reference = high) |  |  |  |
| Low | 0.01 | -0.05 | 0.07 |
| **Family-perceived wealth** (reference = high) |  |  |  |
| Low | 0.10 | 0.00 | 0.21 |
| Medium | 0.03 | -0.02 | 0.08 |
| **Place of residence** (reference = urban) |  |  |  |
| Rural | 0.05 | -0.02 | 0.11 |
| **COVID-19 impact** (reference = no change) |  |  |  |
| Decreased | -0.18* | -0.33 | -0.03 |
| Increased | 0.20* | 0.15 | 0.25 |

Notes. *R^2^* = 0.044; *Wald F* = 16.236; df = 13, 491; *p* < .001; *statistically significant (95% confidence interval does not include 0)

**Table S9** Results of multiple linear regression examining the association between perceived COVID-19 impact on screen time and post-pandemic levels of screen time on weekend

|  |  | **95% Confidence Interval** | |
| --- | --- | --- | --- |
|  | ***B*** | **Lower** | **Upper** |
| **Age (continuous)** | 0.14* | 0.07 | 0.22 |
| **Gender** (reference = female) |  |  |  |
| Male | 0.19* | 0.12 | 0.26 |
| **Weight status** (reference = normal weight) |  |  |  |
| Thinness | 0.09 | -0.18 | 0.36 |
| Overweight | 0.05 | -0.04 | 0.14 |
| Obesity | 0.17* | 0.06 | 0.28 |
| **Parental education** (reference = high) |  |  |  |
| Low | 0.26* | 0.17 | 0.36 |
| Medium | 0.15* | 0.05 | 0.24 |
| **Parental employment** (reference = high) |  |  |  |
| Low | -0.06 | -0.15 | 0.03 |
| **Family-perceived wealth** (reference = high) |  |  |  |
| Low | 0.11 | -0.04 | 0.27 |
| Medium | 0.01 | -0.07 | 0.08 |
| **Place of residence** (reference = urban) |  |  |  |
| Rural | 0.05 | -0.05 | 0.15 |
| **COVID-19 impact** (reference = no change) |  |  |  |
| Decreased | -0.33* | -0.58 | -0.10 |
| Increased | 0.46* | 0.39 | 0.54 |

Notes. *R^2^* = 0.061; *Wald F* = 24.275; df = 13, 494; *p* < .001; *statistically significant (95% confidence interval does not include 0)

**Table S10** Results of multiple linear regression examining the association between perceived COVID-19 impact on learning time and post-pandemic levels of learning time

|  |  | **95% Confidence Interval** | |
| --- | --- | --- | --- |
|  | ***B*** | **Lower** | **Upper** |
| **Age (continuous)** | 0.02 | -0.03 | 0.07 |
| **Gender** (reference = female) |  |  |  |
| Male | -0.07* | -0.11 | -0.02 |
| **Weight status** (reference = normal weight) |  |  |  |
| Thinness | 0.03 | -0.12 | 0.18 |
| Overweight | 0.10* | 0.04 | 0.16 |
| Obesity | 0.05 | -0.01 | 0.12 |
| **Parental education** (reference = high) |  |  |  |
| Low | 0.21* | 0.15 | 0.28 |
| Medium | 0.14* | 0.08 | 0.20 |
| **Parental employment** (reference = high) |  |  |  |
| Low | -0.01 | -0.06 | 0.05 |
| **Family-perceived wealth** (reference = high) |  |  |  |
| Low | 0.08 | -0.02 | 0.17 |
| Medium | 0.03 | -0.02 | 0.08 |
| **Place of residence** (reference = urban) |  |  |  |
| Rural | 0.08* | 0.02 | 0.14 |
| **COVID-19 impact** (reference = no change) |  |  |  |
| Decreased | -0.23* | -0.36 | -0.12 |
| Increased | 0.02 | -0.03 | 0.07 |

Notes. *R^2^* = 0.028; *Wald F* = 7.524; df = 13, 493; *p* < .001; *statistically significant (95% confidence interval does not include 0)

**Table S11** Results of multiple linear regression examining the association between perceived COVID-19 impact on sleep time and post-pandemic levels of sleep time

|  |  | **95% Confidence Interval** | |
| --- | --- | --- | --- |
|  | ***B*** | **Lower** | **Upper** |
| **Age (continuous)** | -0.06* | -0.10 | -0.03 |
| **Gender** (reference = female) |  |  |  |
| Male | -0.04 | -0.07 | 0.00 |
| **Weight status** (reference = normal weight) |  |  |  |
| Thinness | 0.13 | -0.00 | 0.27 |
| Overweight | -0.04 | -0.08 | -0.00 |
| Obesity | -0.13* | -0.19 | -0.08 |
| **Parental education** (reference = high) |  |  |  |
| Low | 0.12* | 0.07 | 0.18 |
| Medium | 0.04 | -0.01 | 0.09 |
| **Parental employment** (reference = high) |  |  |  |
| Low | 0.13 | 0.09 | 0.17 |
| **Family-perceived wealth** (reference = high) |  |  |  |
| Low | -0.02 | -0.10 | 0.05 |
| Medium | 0.01 | -0.03 | 0.04 |
| **Place of residence** (reference = urban) |  |  |  |
| Rural | 0.03 | -0.03 | 0.09 |
| **COVID-19 impact on weekday** (reference = no change) |  |  |  |
| Decreased | -0.12 | -0.28 | 0.05 |
| Increased | -0.06 | -0.12 | 0.00 |
| **COVID-19 impact on weekend** (reference = no change) |  |  |  |
| Decreased | -0.16 | -0.34 | 0.04 |
| Increased | -0.04 | -0.10 | 0.02 |

Notes. *R^2^* = 0.039; *Wald F* = 8,262; df = 15, 494; *p* < .001; *statistically significant (95% confidence interval does not include 0)
